# Deep biomarkers of human aging: Application of deep neural networks to biomarker development

**DOI:** 10.18632/aging.100968

**Published:** 2016-05-18

**Authors:** Evgeny Putin, Polina Mamoshina, Alexander Aliper, Mikhail Korzinkin, Alexey Moskalev, Alexey Kolosov, Alexander Ostrovskiy, Charles Cantor, Jan Vijg, Alex Zhavoronkov

**Affiliations:** ^1^ Pharma.AI Department, Insilico Medicine, Inc, Baltimore, MD 21218, USA; ^2^ Computer Technologies Lab, ITMO University, St. Petersburg 197101, Russia; ^3^ The Biogerontology Research Foundation, Oxford, UK; ^4^ School of Systems Biology, George Mason University (GMU), Fairfax, VA 22030, USA; ^5^ Invitro Laboratory, Ltd, Moscow 125047, Russia; ^6^ Department of Biomedical Engineering, Boston University, Boston, MA 02215, USA; ^7^ Department of Genetics, Albert Einstein College of Medicine, Bronx, NY 10461, USA

**Keywords:** deep learning, deep neural networks, biomarker development, aging biomarkers, human aging, machine learning

## Abstract

One of the major impediments in human aging research is the absence of a comprehensive and actionable set of biomarkers that may be targeted and measured to track the effectiveness of therapeutic interventions. In this study, we designed a modular ensemble of 21 deep neural networks (DNNs) of varying depth, structure and optimization to predict human chronological age using a basic blood test. To train the DNNs, we used over 60,000 samples from common blood biochemistry and cell count tests from routine health exams performed by a single laboratory and linked to chronological age and sex. The best performing DNN in the ensemble demonstrated 81.5 % epsilon-accuracy *r* = 0.90 with *R^2^* = 0.80 and MAE = 6.07 years in predicting chronological age within a 10 year frame, while the entire ensemble achieved 83.5% epsilon-accuracy *r* = 0.91 with *R*^2^ = 0.82 and MAE = 5.55 years. The ensemble also identified the 5 most important markers for predicting human chronological age: albumin, glucose, alkaline phosphatase, urea and erythrocytes. To allow for public testing and evaluate real-life performance of the predictor, we developed an online system available at http://www.aging.ai. The ensemble approach may facilitate integration of multi-modal data linked to chronological age and sex that may lead to simple, minimally invasive, and affordable methods of tracking integrated biomarkers of aging in humans and performing cross-species feature importance analysis.

## INTRODUCTION

Aging is a complex process affecting all biological systems at every level of organization [1, 2]. While many anti-aging interventions have demonstrated life-extending or other geroprotective effects in model organisms, practical limitations continue to hamper translation to the clinic [3]. One problem is that the evaluation of aging changes and possible anti-aging remedies requires a comprehensive set of robust biomarkers [4]. Large-scale longitudinal programs like MARK-AGE [5] have been launched to analyze changes in multiple biomarkers during aging and correlation between biological and chronological age. Several “aging clocks” able to predict human chronological age using various biomarkers have already been proposed. Methylation-based markers such as epigenetic aging clocks (Horvath [6] and Hannum [7]) are currently the most accurate, while transcriptomics [8,9] and metabolomics [10] have shown to be less so. Telomere length is commonly used to measure senescence but has lower predictive ability of human chronological age than IgG N-glycans, immunoglobulin G glycosylated at conservative N-glycation sites [11]. Recent studies show that biomarkers of age-related pathologies could be used to evaluate senescence modifications based on the connection between age-related pathologies at the signaling pathway level [12].

However, most of these biomarkers are not representative of the health state of the entire organism or individual systems and are not easily measured or targeted with known interventions. The common blood biochemistry test is one of the simplest tests used by physicians to examine the health state of patients. While being highly variable in nature, some markers from blood biochemistry are sensitive indicators of various conditions, such as inflammation and even alcoholism, and are approved for clinical use [13, 14].

Machine learning (ML) techniques, such as support vector machines (SVM), are routinely used in biomarker development [15] and rapid increases in labeled data are enabling deep neural networks (DNNs). Methods based on deep architectures have outperformed classical approaches not only in image analysis, but also in solving a wide range of genomics, transcriptomics and proteomics problems [16].

In this study, we apply a deep learning technique for predicting human chronological age that utilizes multiple DNNs stacked into an ensemble and trained on tens of thousands of blood biochemistry samples from patients undergoing routine physical examinations. We then use a custom implementation of the permutation feature importance (PFI) technique [17] to evaluate the relative importance of each blood biochemistry marker to ensemble accuracy. We also analyzed the performance and accuracy of 40 DNN architectures optimized using a variety of optimizers, identified the best DNN, and selected 21 DNNs that cumulatively provided higher accuracy and *R*^2^ as an ensemble than the best DNN in the ensemble.

## RESULTS

To perform this study, we obtained a dataset of 62,419 anonymized blood biochemistry records, where each record consists of a person's age, sex, and 46 standardized blood markers through a collaboration with one of the largest laboratory networks in Russia, Invitro Laboratory, Ltd. We aimed to draw data from a reasonably healthy population. While we did not have access to patient records, we selected only blood tests from routine health checks, avoiding obvious sources of unhealthy patients, such as hospitals, and through statistical analysis omitted blood tests with outliers.

**Figure 1:**
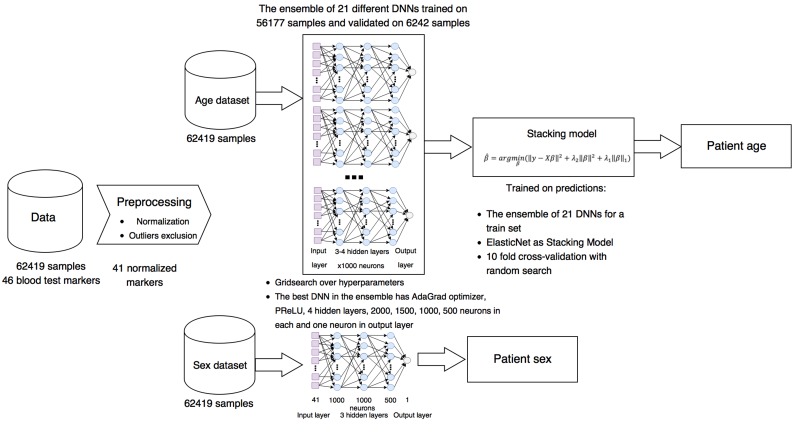
Project pipeline Laboratory blood biochemistry data sets were normalized and cleaned of outliers and some abnormal markers. For biological age prediction, 21 different DNNs with different parameters were combined in ensemble based on ElasticNet model. For biological sex prediction, single DNN were trained.

The generalized project pipeline is depicted in Figure 1. First, we preprocessed the blood test data set, excluding highly biased markers from reference ranges, normalizing them for training the DNNs, and removing outliers (see Methods for details). The resulting data set was split into training and test sets comprised of 56,177 and 6242 samples, respectively. Then 40 different DNNs were trained on 56,177 blood test samples.

Since we treated human age prediction as a regression problem, we used two metrics to estimate the performance of the method: standard coefficient of determination (R^2^) and ε-prediction (epsilon-prediction) accuracy (see Methods for details). When using epsilon-prediction accuracy, the sample is considered correctly recognized if the predicted age is in the range of [true age -ε; true age +ε], where ε controls the level of certainty in the prediction. So if ε = 0, then it is a simple classification accuracy. In this study, we considered ε = 10. The key advantage of using epsilon-prediction accuracy is that it allows cohort analysis without fixed age ranges (e.g. 10-20, 20-30).

**Figure 2:**
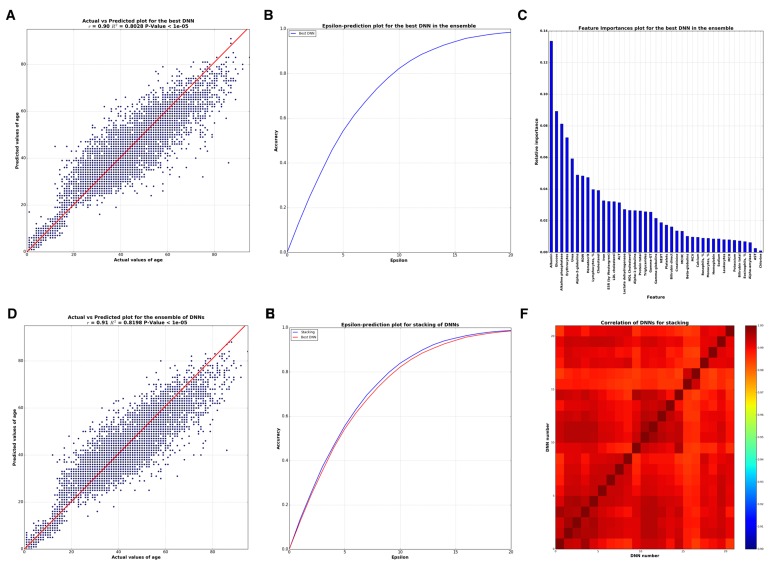
Analysis of best DNN model in the ensemble and the whole ensemble (**A**) Correlation between actual and predicted age values by the best DNN in the ensemble. (**B**) Biological age epsilon-prediction accuracy plot for the best DNN. (**C**) Biological age marker Importance, performed using FPI method. (**D**) Correlation between actual and predicted age values by whole ensemble based on ElasticNet model. (**E**) Biological age epsilon-prediction accuracy plot for the ensemble. (**F**) Heat map for Pearson's correlation coefficients between 40 DNNs. Scale bar colors indicate the sign and magnitude of Pearson's correlation coefficient between predictions of DNNs.

**Figure 3:**
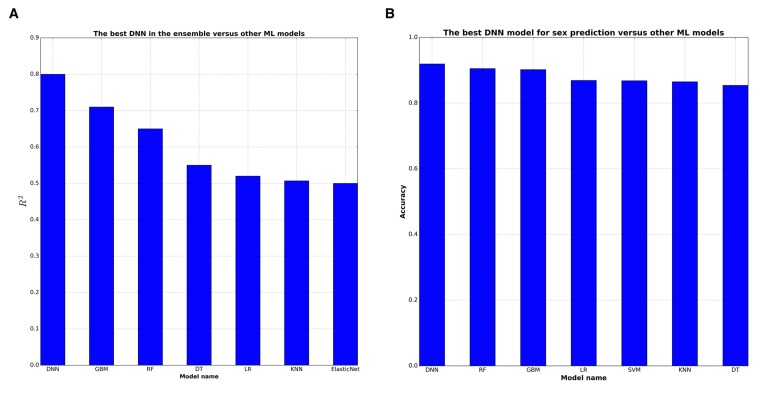
DNNs outperform baseline ML approaches in terms of R^2^ statistics DNN were compared with 7 ML techniques: GBM (Gradient Boosting Machine), RF (Random Forests), DT (Decision Trees), LR (Linear Regression), kNN (k-Nearest Neighbors), ElasticNet, SVM (Support Vector Machines). (**A**) GBM shows the higher 0,72 R^2^ among ML models for biological age prediction. (**B**) All ML models have comparable high R^2^ for biological sex prediction.

The best single DNN performed with 0.80 of R^2^ and 82% within the 10 year frame of epsilon-prediction accuracy (Figure 2A & B). Single DNN outperformed other ML models such as k-Nearest Neighbors, Support Vector Machine, Random Forests, Gradient Boosting Machine, etc (Figure 3 & B).

**Figure 4:**
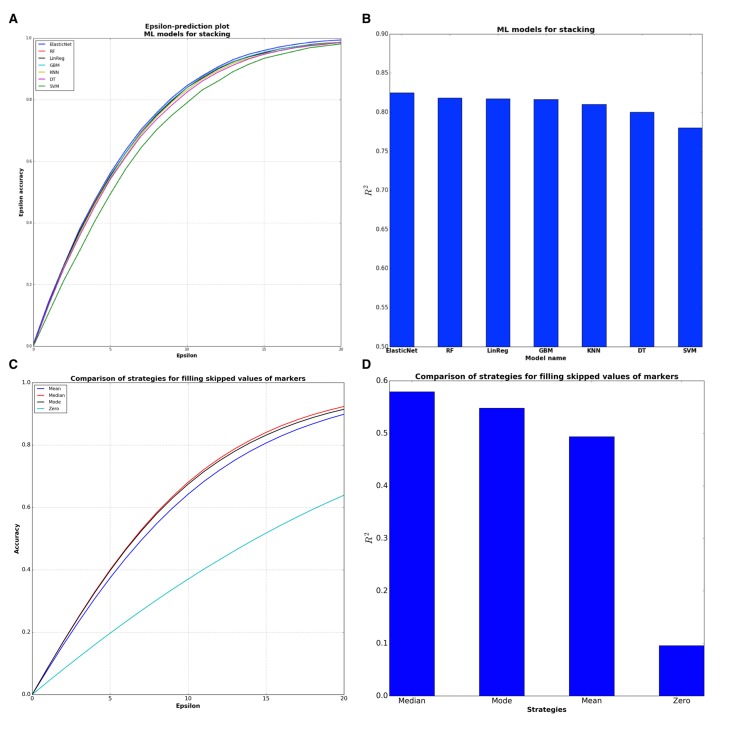
Comparison of sub-models for stacking ensemble and evaluation of filling strategies (**A**) ElasticNet model has the higher epsilon-prediction accuracy among the stacking models. (**B**) ElasticNet is the best model for stacking from the point of R^2^ statistics. (**C**) Median filling strategy has higher epsilon-prediction accuracy than other strategies. Median filling strategy shows 64,5 % epsilon accuracy within 10 years frame. (**D**) Median filling strategy is better from the point of R^2^ statistics.

To further increase the coefficient of determination and accuracy of predictions, we combined these single DNNs into an ensemble based on the stacked generalization (Stacking) technique [18]. Stacking is a method that fits some ML models on the predictions of other models, in our case on the predictions of DNNs. Model selection was performed with 10 fold cross-validation and with the random search strategy for finding the best hyperparameters for considered models. The experiments with Stacking models showed (Figure 4A & B) that the best ML model was ElasticNet.

To successfully combine the predictions of DNNs into the Stacking ensemble model, the predictions of DNNs should closely approximate the target variable and differ from one another, or be less correlated. To achieve this, DNNs should be trained with different hyperparameters, varying in the number of layers, counts of neurons in each layer, activation functions, regularization techniques, etc. We investigated 40 DNNs, each unique in terms of hyperparameters. Pearson correlations of these DNNs are presented in a heat map on Figure 2F, showing a high degree of similarity among many of the networks regarding predictions (r approaching 1) but also some major distinctions.

To determine how many of these trained DNNs were necessary for constructing the Stacking ensemble model, we performed an iterative process of adding each DNN's predictions vector into the ensemble. Two iterative strategies were employed: adding predictions by decreasing R^2^ of each network, i.e. adding better networks considering R^2^ earliest in the ensemble, and increasing the correlation between DNNs, i.e. adding less correlated networks first. The results of this assay are presented in Figure S2. Both strategies showed that no more than 21 DNNs were needed in the ensemble. The ensemble resulting from distinguishing the correlations of DNNs and ordering the addition of DNNs into the ensemble demonstrated R^2^=0.82 and 83,5% within a 10 year frame of epsilon-prediction accuracy (Figure 2D & E).

We compared our deep-learned predictor with several published epigenetics and transcriptomics markers of human age. Surprisingly, despite the fact that we used only blood biochemistry data with 41 values for each patient, our biomarker outperformed blood transcriptomics biomarkers presented by Peters et al with R^2^=0,6 for the best model [8]. Due to the nature of the data, epigenetics markers show a stronger correlation with chronological age, with R^2^=0,93 for Horvath's methylation clock and R^2^=0,89 for the Hannum et methylation clock [6, 7].

### Marker importance

In order to analyze the importance of blood test markers via neural networks, some wrapper feature (selection) importances approaches are required. We used a modification of the Permutation Feature Importance (PFI) method (see Methods for details). By applying this method, one receives a list sorted by the importance of markers via DNN. This technique has two benefits: 1) it is native and simple to interpret and 2) as other wrapper methods it relies on DNN performance, which in this case is better than other ML models, thus produces more robust and meaningful features. Marker importance analysis by PFI method, the results of which are presented in Figure 2C, reveals the five important markers: albumin, glucose, alkaline phosphatase, urea, and erythrocytes.

### Top features

**Figure 5:**
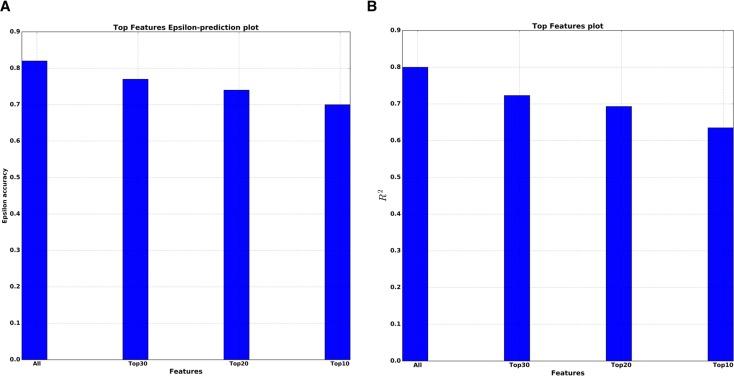
Top features analysis (**A**) Dependence of the epsilon-prediction accuracy from the number of features. (**B**) Dependence of R^2^ statistics from the number of features.

We also performed so-called top features analysis, which answers how the performance of a single DNN will decrease as the number of markers used in the model decreases. To select the smaller number of markers for training the DNN, the sorted list of all PFI scores is used. The results of this analysis for both R^2^ and epsilon-prediction accuracy are presented on Figure 5A & B. For the top 10 features by PFI, the DNN got R^2^=0.63 and 70% of 10 year frame epsilon-accuracy prediction. In practical terms, the fact that this drop in performance was so small supports the top 10 markers received by PFI as robust and reliable features for predicting age.

### Use case

To make this deep network ensemble available to the public, we placed our system online (www.Aging.AI), allowing any patient with blood test data to predict their age and sex. In order to validate our approach, we collected the blood biochemistry reports that were uploaded on the site from 25 January to 15 March 2016. The total number of collected reports with indicated real age was 1,563 samples. Many users expressed no desire to specify all 41 parameters of the blood test, so we added an option to enter only the 10 most important markers. The average number of missing values provided by the volunteer testers was 18.5 markers per person. There are several strategies for filling skipped values, including zero, mean, mode and median over all values of each marker. Evaluation of these 4 strategies on the aging.ai data showed that median filling strategy has the best performance in terms of both R^2^ and epsilon-prediction accuracy (Figure 4C & D).

Aging.AI provides a proof of concept for a simple and inexpensive blood-based predictor of chronological age, which may be used for speculate on the biological age of the patient. However, it has many limitations. When it comes to developing predictors using deep neural networks, one of the major difficulties is building large data sets. In this study we were constrained by the limited number of features available to us in large numbers of blood test results. Some of the features, for example globulin fractures, are no longer frequently used in diagnostic medicine and are excluded from the newer standard tests. However, these features were present in historical tests available in large numbers and were used for training.

## DISCUSSION

Aging is a complex process and occurs at different rates and to different extents in the various organ systems, including respiratory, renal, hepatic, and metabolic [19, 20]. The analysis of relative feature importance within the DNNs helped deduce the most important features that may shed light on the contribution of these systems to the aging process, ranked in the following order: metabolic, liver, renal system and respiratory function. The five markers related to these functions were previously associated with aging and used to predict human biological age [21, 22]. Another interesting finding was the extraordinarily high importance of albumin, which primarily controls the oncotic pressure of blood. Albumin declines during aging and is associated with sarcopenia [23]. The second marker by relative importance is glucose, which is directly linked to metabolic health. Cardiovascular diseases associated with diabetes mellitus are major causes of death within the general population [24].

Our approach of using an ensemble of DNNs outperformed other ML models in terms of R^2^ and epsilon-prediction accuracy (Figure 3A & B). Application of DNNs uncovered complex nonlinear interactions between markers resulting in robust ensemble performance. This ensemble may also be expanded with DNNs trained on different sources and types of biological data allowing for complex multi-modal markers to be created and relative contributions of each input analyzed.

Current and future directions of this work include adding other sources of features including transcriptomic and metabolomics markers from blood, urine, individual organ biopsies and even imaging data as well as testing the system using data from patients with accelerated aging syndromes, multiple diseases and performing gender-specific analysis. Similar tests may be performed by research teams working on rare diseases or working with athletic groups by using http://www.Aging.AI system or contacting the authors to perform a high-throughput analysis. Developing similar systems for model organisms and performing PFI analysis may help perform cross-species analysis and of the relative importance of individual markers and organ systems in predicting chronological and biological age.

## MATERIALS AND METHODS

### Data

Anonymized statistical data of human blood tests was kindly provided by an independent laboratory, Invitro (http://www.Invitro.ru). No patient records were used in the study. In total, the data contains 62419 records where each record consists of person's age and 46 standardized blood markers, such as Glucose, Cholesterol, Alpha-1-globulins, etc. (Table S1) Histograms of human age for training sets and descriptive statistics of top 10 blood markers used in the research are depicted in the Figure S1A.

One can see from the Figure S1B that minimum and maximum values of each marker are far distributed from their normal range values. This distribution reflects patients' tendencies to self-report symptoms and test their health with professional health-care services only in complex cases, which affects their health condition and thus test results [25]. Moreover, we found that there were no patients that could be considered as healthy and who have blood test values within a reference range. The most frequently abnormal markers in the distribution were white blood cell count markers: basophils, abs., eosinophils, abs., lymphocytes abs. monocytes, abs, neutrophils, abs. These types of test provide the total number (absolute number, abs.) of white blood cells in blood microliter. Here, this routine analysis was conducted using a hematology automated analyzer, which counts cells precisely with low error rate [26]. In this case, these aberrant values of markers are more likely linked to the major function of white blood cells; immune function, infections, allergies, smoking [27] or even sleep duration [28] could affect the rate of white blood cells. Additionally, recent studies show a connection between metabolic diseases such as diabetes and range of white blood cells [29, 30]. For this reason, levels of basophils, eosinophils, lymphocytes, monocytes and neutrophils are extremely variable in the general population. To prevent DNN predictions from being highly biased with respect to abnormal ranges of blood markers, we excluded these 5 markers. Processed data was presented in a tabular format of 62419 rows and 42 columns (age and sex + 41 markers).

Then, specifically for training deep neural network, we normalized all blood markers to 0-1 range by using the formula:

$$X_{0-1}=\frac{X-X_{\text{min}}}{X_{\text{max}}-X_{\text{min}}}$$

where *X* is the origin values of each blood marker, *X_min_* and *X_max_* are its minimum and maximum, respectively and *X_0–_* is the marker within 0-1 range.

We split the data to the training and test sets with 90/10 ratio. Thus, the size of training and test sets were 56177 and 6242 samples, respectively. The DNN was built by adjusting its hyperparameters (such as a number of layers, activation function, etc.) on the training set and measuring the performance of the trained neural network on the test set. The comparison of performances of 6 best DNNs with different values of hyperparameters is depicted on Table S1. All experiments were conducted on Nvidia Tesla K80 graphics processing unit.

There are two reasons why in the study we treated the prediction of human age as a regression problem: 1) age has natural order, so it is an order variable and 2) one may be interested in the difference in values of the markers with difference in ages, which is the natural way to perform the analysis of marker influence. In this case, it was better to use regression instead of classification methods.

So, in all evaluations 4 metrics were measured:

*r*, which is a Pearson's correlation coefficient defined as: $r=\frac{\sum_{i=1}^{N}(x_{i}-\bar{x})(y_{i}-\bar{y})}{\sqrt{\sum_{i=1}^{N}{\left(x_{i}-\bar{x}\right)}^{2}}\sqrt{\sum_{i=1}^{N}{\left(y_{i}-\bar{y}\right)}^{2}}}$; where *x_i_* is real value and $\bar{x}$ is the mean of *x*, *y_i_* is predicted value and $\bar{y}$ is the mean of *y*, and *N* is number of samples.
*R^2^*, which is a standard coefficient of determination defined as: $R^{2}=\frac{\sum_{i=1}^{N}{\left(y_{i}-f_{i}\right)}^{2}}{\sum_{i=1}^{N}{\left(y_{i}-\bar{y}\right)}^{2}}$; where *y_i_* is the real value, *f_i_* is the predicted value and $\bar{y}$ is the mean of *y*.
Mean absolute error (MAE), which is defined as $\text{MAE}=\frac{1}{N}\overset{N}{\underset{i=1}{\sum }}\left|f_{i}-y_{i}\right|$; where *f_i_* is a prediction of the model, *y_i_* is a true value and N is a number of samples.
*ε*-prediction accuracy defined as: $\varepsilon -\text{prediction}=\frac{\sum_{i=1}^{N}1_{A}(f_{i})}{N}$; where A is [*y_i_* − *ε*; *y_i_* + *ε*]; *y_i_* is the real value, *f_i_* is the predicted value and *ε* is a parameter that controls the range of correctness of predictions. So for example if *ε* is 10 and the true value of age is 45 the deep neural network correctly recognized sample if it is in the [35, 55] range.

### Feature importance method

The idea behind the algorithm stemmed from the feature randomization technique used in Random Forest (RF) [31]. PFI computes significance scores for all features by determining the accuracy of a model to random permutations of the values of those feature variables. The main underlying assumption is that permuting the values of important features results in a more significant reduction in a model's performance compared to the effect of less important ones. But when cross-validation is not performed, one should improve the robustness of the method.

To do this, we shuffled each feature *k* times and then computed the average PFI score for the feature, concretely the PFI score for one feature is defined as follows:

$PFI_{\text{feature}}=R_{\text{total}}^{2}-\frac{1}{N}\overset{N}{\underset{i=1}{\sum }}R_{\text{shuffle}}^{2}$; where $R_{\text{total}}^{2}$ is a total *R^2^* for the model without any permutations and $R_{\text{shuffle}}^{2}\;$ is a *R^2^* for the model with permutated feature permutated feature and *K* is a parameter that controls how many times the feature is permutated.

Note that PFI is a wrapper method, so it would significantly depend on applying ML model, but because DNNs show better performance than other ML models, it was suitable for the problem.

### Architecture of DNN

We used simple feed-forward neural networks trained with the standard backpropagation algorithm as our deep (more than 3 layers) learning models. For each DNN in the resulting ensemble, multiple hyperparameters were adjusted, including the number of hidden layers, the number of neurons in each layer, choice of activation function, choice of optimization method, and regularization techniques. The table with experiments of different hyperparameters for the DNNs are presented in Table S1.

The best DNN in the ensemble had 5 hidden layers with 2000, 1500, 1000, 500, and 1 neurons in each, respectively. The last layer, with one neuron, corresponds to regression output. The optimization loss function was simple mean squared error (MSE) with regularization terms. The DNN used PReLU activation function [32] in each layer, AdaGrad [33] as optimizer of the loss function, Dropout [34] with probability of 0.2 after each layer, and l2 weight decay [35]. To further cope with over fitting and make more stable convergence of models, we used Batch normalization technique [36] after the first 2 layers.

## SUPPLEMENTARY DATA FIGURES AND TABLES



## Abbreviations

ML: Machine Learning
SVM: Support Vector Machine
DNN: Deep Neural Network
PFI: Permutation Feature Importance
RF: Random Forests
GBM: Gradient Boosting Machine
kNN: k-Nearest Neighbors
DT: Decision Trees
LR: Linear Regression
