# Supplementary material for: Deep biomarkers of human aging: Application of deep neural networks to biomarker development
Source: Aging (Albany NY). 2016 May 18;8(5):1021–30. doi: 10.18632/aging.100968 (PMC4931851; doi:10.18632/aging.100968)
Supplement: Supplementary file 1 [file aging-08-1021-s001.pdf]

SUPPLEMENTARY DATA

A

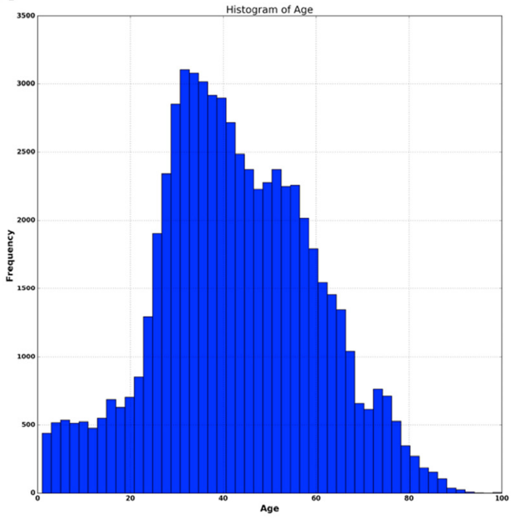

B

Descriptive statistics for top 10 markers by PFI method

Test name	Mean	Std	Q25%	Q50%	Q75%	Min	Max
Albumin	43.57	4.03	41.61	43.96	46.14	1.00	59.25
Erythrocytes	4.76	0.51	4.44	4.75	5.09	0.79	9.25
Glucose	5.57	1.26	4.99	5.36	5.80	0.35	32.24
Alkaline phosphatase	85.96	78.52	56.00	70.00	89.00	1.00	4337.00
Hematocrit	40.89	4.43	38.20	40.90	43.90	8.00	66.00
Urea	5.17	2.21	3.90	4.90	6.00	0.70	84.10
RDW	13.71	1.97	12.70	13.20	13.90	1.00	44.20
Cholesterol	5.48	1.27	4.58	5.38	6.26	1.00	20.19
Alpha-2-globulins	7.06	1.31	6.18	6.86	7.70	1.00	20.17
Lymphocytes	35.48	10.07	29.00	35.00	41.30	0.00	98.00

Figure S1. (**A**) Histogram of age distribution. (**B**) Table of descriptive statistic for top 10 markers.

Table S1. Table of hyperparameters. The best DNN in the ensemble has AdaGrad optimizer, PReLU activation function and 4 hidden layers with 2000, 1500, 1000, 500 neurons respectively and got 0.803 of R^2.

DNN architecture. Hidden units	Additional parameters. Activation function/Optimizer		
	ReLU/AdaDelta	ReLU/AdaGrad	PReLU/AdaGrad
1000-1000-500	0.742	0.77	0.773
1000-1000-1000-500	0.745	0.782	0.792
1000-1000-1000-1000	0.75	0.784	0.785
1500-1500-1500-1500	0.754	0.791	0.795
2000-1500-1000-500	0.755	0.792	**0.805**
2500-2500-2500-2500	0.745	0.775	0.781

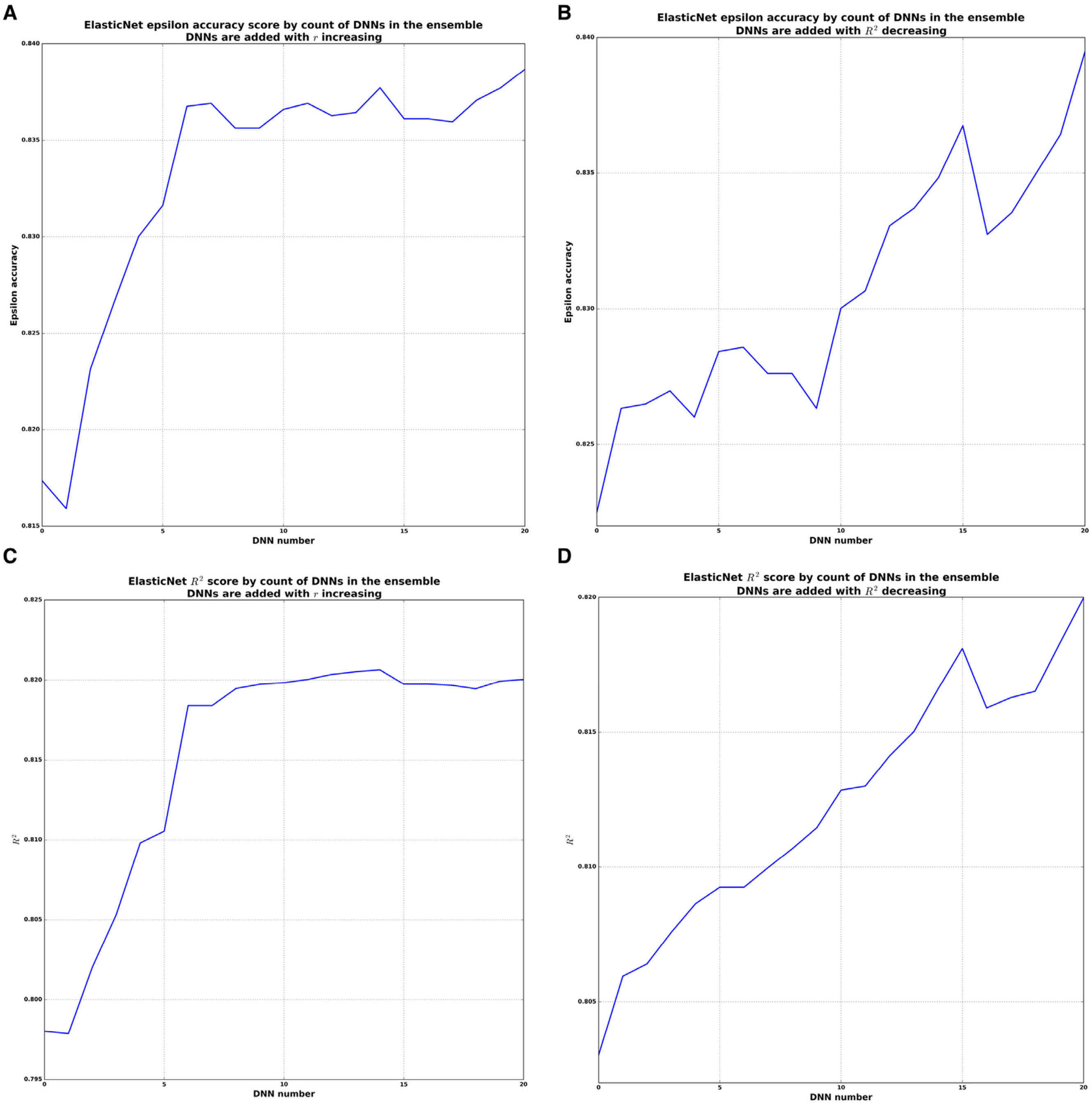

Figure S2. Analysis of the ensemble based on ElasticNet model. (**A**) Epsilon accuracy plot for constructing ensemble where DNNs are added with *r* increasing. (**B**) Epsilon accuracy plot for constructing ensemble where DNNs are added with R^2 decreasing. (**C**) R^2 plot for constructing ensemble where DNNs are added with r increasing. (**D**) R^2 plot for constructing ensemble where DNNs are added with R^2 decreasing,

Powell J, et al. The transcriptional landscape of age in human peripheral blood. Nat. Commun. 2015; 6:8570.

9. Nakamura S, Kawai K, Takeshita Y, Honda M, Takamura T, Kaneko S, et al. Identification of blood biomarkers of aging by transcript profiling of whole blood. Biochem. Biophys. Res. Commun. 2012; 418:313–318.

10. Menni C, Kastenmüller G, Petersen AK, Bell JT, Psatha M, Tsai P-C, et al. Metabolomic markers reveal novel pathways of ageing and early development in human populations. Int. J. Epidemiol. 2013; 42:1111–1119.

11. Krištić J, Kri ti J, Vu kovi F, Menni C, Klari L, Keser T, et al. Glycans Are a Novel Biomarker of Chronological and Biological Ages. J. Gerontol. A Biol. Sci. Med. Sci. 2013; 69:779–789.

12. Aliper AM, Csoka AB, Buzdin A, Jetka T, Roumiantsev S, Moskalev A, et al. Signaling pathway activation drift during aging: Hutchinson-Gilford Progeria Syndrome fibroblasts are comparable to normal middle-age and old-age cells. Aging (Albany NY). 2015; 7:26–37. doi: 10.18632/aging.100717.

13. Kaysen GA. Biochemistry and biomarkers of inflamed patients: why look, what to assess. Clin. J. Am. Soc. Nephrol. 2009; 4 Suppl 1:S56–63.

14. Peterson K. Biomarkers for alcohol use and abuse--a summary. Alcohol Res. Health. 2004; 28:30–37.

15. Libbrecht MW, Noble WS. Machine learning applications in genetics and genomics. Nat. Rev. Genet. 2015; 16:321–332.

16. Mamoshina P, Polina M, Armando V, Evgeny P, Alex Z. Applications of Deep Learning in Biomedicine. Mol. Pharm. Internet. 2016; Available from: http://dx.doi.org/10.1021/acs.molpharmaceut.5b00982.

17. Altmann A, Toloşi L, Sander O, Lengauer T. Permutation importance: a corrected feature importance measure. Bioinformatics. 2010;26:1340–1347.

18. Wolpert DH. Stacked generalization. Neural Netw. 1992;5:241–259.

19. Levine ME. Modeling the rate of senescence: can estimated biological age predict mortality more accurately than chronological age? J. Gerontol. A Biol. Sci. Med. Sci. 2013; 68:667–674.

20. Cheng S, Larson MG, McCabe EL, Murabito JM, Rhee EP, Ho JE, et al. Distinct metabolomic signatures are associated with longevity in humans. Nat. Commun. 2015; 6:6791.

21. Cohen AA, Milot E, Li Q, Bergeron P, Poirier R, Dusseault-Bélanger F, et al. Detection of a novel, integrative aging process suggests complex physiological integration. PLoS One. 2015; 10:e0116489.

22. Park J, Cho B, Kwon H, Lee C. Developing a biological age assessment equation using principal component analysis and clinical biomarkers of aging in Korean men. Arch. Gerontol. Geriatr. 2009; 49:7–12.

23. Visser M, Kritchevsky SB, Newman AB, Goodpaster BH, Tylavsky FA, Nevitt MC, et al. Lower serum albumin concentration and change in muscle mass: the Health, Aging and Body Composition Study. Am. J. Clin. Nutr. 2005; 82:531–537.

24. World Health Organization. Health in 2015: from MDGs, Millennium Development Goals to SDGs, Sustainable Development Goals. WHO (World Health Organization); 2015.

25. Elnegaard S, Andersen RS, Pedersen AF, Larsen PV, Søndergaard J, Rasmussen S, et al. Self-reported symptoms and healthcare seeking in the general population--exploring "The Symptom Iceberg." BMC Public Health. 2015; 15:685.

26. Devreese K, De Logi E, Francart C, Heyndrickx B, Philippé J, Leroux-Roels G. Evaluation of the automated haematology analyser Sysmex NE-8000. Eur. J. Clin. Chem. Clin. Biochem. 1991; 29:339–345.

27. Hansen LK, Grimm RH, Neaton JD. The Relationship of White Blood Cell Count to Other Cardiovascular Risk Factors. Int. J. Epidemiol. 1990; 19:881–888.

28. Boudjeltia KZ, Faraut B, Stenuit P, Esposito MJ, Dyzma M, Brohée D, et al. Sleep restriction increases white blood cells, mainly neutrophil count, in young healthy men: a pilot study. Vasc. Health Risk Manag. 2008; 4:1467–1470.

29. Babio N, Ibarrola-Jurado N, Bulló M, Martínez-González MÁ, Wärnberg J, Salaverría I, et al. White blood cell counts as risk markers of developing metabolic syndrome and its components in the PREDIMED study. PLoS One. 2013; 8:e58354.

30. Twig G, Afek A, Shamiss A, Derazne E, Tzur D, Gordon B, et al. White blood cells count and incidence of type 2 diabetes in young men. Diabetes Care. 2013; 36:276–282.

31. Breiman L, Leo B, Michael L, John R. Random Forests: Finding Quasars. Statistical Challenges in Astronomy. 2001; 243–254.

32. He K, Kaiming H, Xiangyu Z, Shaoqing R, Jian S. Delving Deep into Rectifiers: Surpassing Human-Level Performance on ImageNet Classification. 2015 IEEE International Conference on Computer Vision (ICCV) Internet. 2015. Available from: http://dx.doi.org/10.1109/iccv.2015.123

33. Duchi J, Hazan E, Singer Y. Adaptive subgradient methods for online learning and stochastic optimization. J. Mach. Learn. Res. 2011;12:2121–2159.

34. Srivastava N, Hinton G, Krizhevsky A, Sutskever I, Salakhutdinov R. Dropout: A simple way to prevent neural networks from overfitting. J. Mach. Learn. Res. 2014; 15:1929–1958.

35. Moody J, Hanson S, Krogh A, Hertz JA. A simple weight decay can improve generalization. Adv. Neural Inf. Process. Syst. 1995; 4:950–957.

36. Ioffe S, Szegedy C. Batch normalization: Accelerating deep network training by reducing internal covariate shift. arXiv preprint. 2015; arXiv:1502.03167.
